# Comparative genomic analysis of a multiple antimicrobial resistant enterotoxigenic *E. coli* O157 lineage from Australian pigs

**DOI:** 10.1186/s12864-015-1382-y

**Published:** 2015-03-10

**Authors:** Ethan Wyrsch, Piklu Roy Chowdhury, Sam Abraham, Jerran Santos, Aaron E Darling, Ian G Charles, Toni A Chapman, Steven P Djordjevic

**Affiliations:** The ithree institute, University of Technology Sydney, P.O. Box 123, Broadway, NSW 2007 Australia; NSW Department of Primary Industries, Elizabeth Macarthur Agricultural Institute, Private Bag 4008, Narellan, NSW 2567 Australia; School of Animal and Veterinary Sciences, University of Adelaide, Adelaide, South Australia 5371 Australia

**Keywords:** Enterotoxigenic *Escherichia coli*, Diarrhoeal disease, Swine, Porcine, O157

## Abstract

**Background:**

Enterotoxigenic *Escherichia coli* (ETEC) are a major economic threat to pig production globally, with serogroups O8, O9, O45, O101, O138, O139, O141, O149 and O157 implicated as the leading diarrhoeal pathogens affecting pigs below four weeks of age. A multiple antimicrobial resistant ETEC O157 (O157 SvETEC) representative of O157 isolates from a pig farm in New South Wales, Australia that experienced repeated bouts of pre- and post-weaning diarrhoea resulting in multiple fatalities was characterized here. Enterohaemorrhagic *E. coli* (EHEC) O157:H7 cause both sporadic and widespread outbreaks of foodborne disease, predominantly have a ruminant origin and belong to the ST11 clonal complex. Here, for the first time, we conducted comparative genomic analyses of two epidemiologically-unrelated porcine, disease-causing ETEC O157; *E. coli* O157 SvETEC and *E. coli* O157:K88 734/3, and examined their phylogenetic relationship with EHEC O157:H7.

**Results:**

O157 SvETEC and O157:K88 734/3 belong to a novel sequence type (ST4245) that comprises part of the ST23 complex and are genetically distinct from EHEC O157. Comparative phylogenetic analysis using PhyloSift shows that *E. coli* O157 SvETEC and *E. coli* O157:K88 734/3 group into a single clade and are most similar to the extraintestinal avian pathogenic *Escherichia coli* (APEC) isolate O78 that clusters within the ST23 complex. Genome content was highly similar between *E. coli* O157 SvETEC, O157:K88 734/3 and APEC O78, with variability predominantly limited to laterally acquired elements, including prophages, plasmids and antimicrobial resistance gene loci. Putative ETEC virulence factors, including the toxins STb and LT and the K88 (F4) adhesin, were conserved between O157 SvETEC and O157:K88 734/3. The O157 SvETEC isolate also encoded the heat stable enterotoxin STa and a second allele of STb, whilst a prophage within O157:K88 734/3 encoded the serum survival gene *bor*. Both isolates harbor a large repertoire of antibiotic resistance genes but their association with mobile elements remains undetermined.

**Conclusions:**

We present an analysis of the first draft genome sequences of two epidemiologically-unrelated, pathogenic ETEC O157. *E. coli* O157 SvETEC and *E. coli* O157:K88 734/3 belong to the ST23 complex and are phylogenetically distinct to EHEC O157 lineages that reside within the ST11 complex.

**Electronic supplementary material:**

The online version of this article (doi:10.1186/s12864-015-1382-y) contains supplementary material, which is available to authorized users.

## Background

Enterotoxigenic *Escherichia coli* (ETEC) are a leading cause of neonatal, pre-weaning and post-weaning diarrhoea (PWD) in pigs. Significant economic losses are incurred as a result of mortalities, reduced growth rates in survivors and medication costs. Antimicrobials have been used to treat infections but the emergence of multiple drug resistant variants poses a serious challenge in controlling ETEC infections in swine production. Dietary zinc supplements, phage therapy, probiotics, antibody dietary formulations and breeding programs to generate swine stock expressing gut epithelial receptors that are not recognised by ETEC fimbriae all represent alternate strategies used by swine producers to control ETEC [1], however these pathogens continue to pose a constant threat to farms.

ETEC serogroups O8, O9, O45, O101, O138, O139, O141, O149 and O157 are implicated in diarrhoeal disease during pig development [2-4]. Porcine enterotoxigenic *E. coli* O157 are associated with disease of the small intestine and mediate neonatal, pre- and post-weaning diarrhoea in piglets. This is in contrast to the mode of pathogenesis displayed by EHEC O157, which is associated with food-borne human illness causing diarrhoea and enterohaemorrhagic disease of the large intestine [5-7]. Multiple evolutionary lineages of *E. coli* O157 [8] with different lineages encoding variants of the H-antigen genes, and two distinct *rfbE* alleles within the O157-antigen biosynthesis gene cluster, have been described. In addition, reports of serogroup O157 isolates as ETEC [9] and EPEC [10,11] is indicative of a wider evolutionary range of pathogenic *E. coli* O157, sourced from multiple hosts including humans, pigs and cattle.

Features separating porcine ETEC from other diarrhoeagenic *E. coli* include the presence of the pig-specific fimbrial adhesins F4 (K88), F5, F6, F18 and F41 that facilitate adherence to porcine gut epithelium and toxins including the heat-stable enterotoxins STa and STb, and a heat-labile enterotoxin (LT) that induce diarrhoea [1,12]. Molecular epidemiological studies indicate that other putative virulence genes are also expressed by ETEC causing ND and PWD, including EAST1 toxin, haemolysins, autotransporters, outer membrane proteins, siderophores and additional iron acquisition factors [2,13,14]. Many putative virulence genes are present only in subsets of clinical ETEC isolates suggesting that different virulence gene combinations may contribute to the range of clinical symptoms observed in ETEC infections. Additionally, ETEC readily acquire virulence and antimicrobial resistance genes by lateral gene transfer and strain variants containing new combinations of virulence and antimicrobial resistance genes are continually evolving globally [13,15-18].

In 2008 a piggery in Australia sustained major economic losses from an outbreak of pre- and post-weaning diarrhoea caused by enterotoxigenic *Escherichia coli* (ETEC) with an O157 serogroup. A representative of the ETEC outbreak is isolate *E. coli* O157 SvETEC. *E. coli* O157 SvETEC caused pre- and post-weaning diarrhoea in pigs between 7–15 days of age, affecting farrowing as well as weaner sheds. Severely affected pigs died suddenly with little or no diarrhoea. Gilts were shown to shed the pathogen but remained free of symptoms during the outbreak. Various antimicrobial and management strategies were implemented and all failed to control the ETEC infection in the pig herd. The outbreak resulted in a significant loss of piglets, over a long duration of time and over multiple sheds. Eventually all stock were removed, pens cleaned and restocked. We present here a comparative genomic analyses of O157 SvETEC with a historical ETEC O157 isolate from Australia (O157:K88 734/3) sampled in the 1990s from a clinical neonatal diarrhoea specimen [4,19].

In this study, a combination of bioinformatics and genome sequencing methodologies was used to perform a comparative and phylogenetic analysis of strains O157 SvETEC and O157:K88 734/3. These are the first ETEC O157 genomes deposited in public databases. Our analyses provide insight into the evolution of ETEC O157 isolates in Australian swine populations revealing that they are phylogenetically distinct to other *E. coli* isolates of serogroup O157. We identified a suite of putative virulence factors, antimicrobial resistance genes and mobile genetic elements in the two porcine O157 isolates. In addition, we highlight novel variability between these two related pathogens.

## Methods

### Strains, isolation, culture conditions and serogrouping

Isolate O157 SvETEC (previously ETEC 95 [19]) was isolated in 2008 from the faeces of an affected piglet from a commercial farm in New South Wales (Australia) that experienced repeated bouts of pre- and post-weaning diarrhoea and high mortality. Isolate O157:K88 734/3 (previously ETEC 24 [19]) was sampled in the 1990s from a clinical submission of common neonatal diarrhoea. Isolates were initially characterised at the NSW Department of Primary Industries Elizabeth Macarthur Agricultural Institute (EMAI) in Menangle [4,13,19]. The strains were sent to the ithree institute at the University of Technology Sydney (UTS), as stab cultures and thereafter regularly cultured in LB broth supplemented with ampicillin (50 μg ml^−1^) with shaking at 200 rpm at 37°C for approximately 16 hours.

### Antimicrobial resistance phenotyping

At EMAI, strains were screened against 18 antimicrobial agents by disc diffusion using the calibrated dichotomous susceptibility (CDS) test Australia, as reported previously [4]. The following antimicrobials were tested: ampicillin (25 μg), amoxicillin/clavulanate (60 μg), ticarcillin/clavulanic acid (85 μg), cefalexin (100 μg), cefoxitin (30 μg), cefotaxime (5 μg), cefepime (10 μg), nalidixic acid (30 μg), ciprofloxacin (2.5 μg), imipenem (10 μg), sulphafurazole (300 μg), trimethoprim (5 μg), tetracycline (10 μg), apramycin (15 μg), neomycin (30 μg), gentamicin (10 μg), azithromycin (15 μg) and chloramphenicol (30 μg).

### Genomic DNA extraction

Sequencing quality gDNA was extracted from 2 mL of each overnight culture using a DNeasy Blood and Tissue Kit (Qiagen) following manufacturer’s recommendations.

### Whole genome sequencing, assembly, annotation and phylogenetic analysis

Sequencing was performed at the UTS in-house Next Generation Sequencing facility using a bench top Illumina MiSeq® sequencer and MiSeq® V3 chemistry. Sequencing libraries were prepared with 0.5 ng of gDNA following the manufacturer’s protocol for the Nextera® XT library preparation kit (Illumina). Sequencing with the MiSeq® sequencer generated 250 nucleotide (nt) long paired end reads of the libraries representing each sample. The quality of the sequence reads was assessed using a locally downloaded version (0.10.1) of FastQC (http://www.bioinformatics.babraham.ac.uk/projects/fastqc/) software and assembled using the A5-miseq *de novo* assembly pipeline [20] revised to process reads up to 500 nt long [21]. Scaffolds over 1000 nt in length were included in the whole genome sequence analysis. Whole Genome Shotgun sequences were deposited at DDBJ/EMBL/GenBank under the accession numbers JPPP00000000 for *E. coli* O157 SvETEC and JPQX00000000 for *E. coli* O157:K88 734/3. A preliminary annotation of each genome was generated using the automated annotation software RAST [22] and the annotation of antimicrobial resistance genes was performed using the Resistance Gene Identifier (RGI) Version 2 on the Comprehensive Antibiotic Resistance Database website [23]. Individual genes of interest, including those annotated by RAST and the RGI, were manually interrogated using NCBI’s BLASTn and BLASTp tools. Insertion sequences (IS) and open reading frames (ORFs) were identified using the online tools IS Finder (https://www-is.biotoul.fr//) and ORF Finder (http://www.ncbi.nlm.nih.gov/gorf/gorf.html) respectively. Phage associated gene clusters within the genomic scaffolds were initially identified using the PHAST [24] server. Genomic scaffolds with positive PHAST hits were further verified to be phage-associated in the RAST annotation output and using BLASTn and BLASTp analysis.

An alignment of phylogenetic marker genes was constructed using PhyloSift [25] and a tree was then inferred using FastTree2 [26]. The publicly available FastTree2 software is unable to resolve branches in the phylogeny shorter than 1×10^−5^ substitutions per site. Our dataset appeared to have several short branches; therefore we modified the FastTree2 software to improve short branch resolution and applied it to our dataset. The output was visualised in FigTree v1.4.2 (http://tree.bio.ed.ac.uk/software/figtree/). The O157 SvETEC and O157:K88 734/3 genomes were analysed alongside 40 complete *E. coli* genomes, 3 complete *Shigella* spp. genomes, 33 draft *E. coli* O157 genomes and 2 draft *E. coli* APEC O78 genomes from the NCBI GenBank database. The *Klebsiella pneumonia* 342 and *Salmonella enterica* subsp. *enterica* serovar Heidelberg str. 41578 genome sequences were included in this analysis as out-groups to confirm the validity of the method; however these sequences were removed from the final phylogenetic tree to facilitate visualizing the fine-scale relationships among *E. coli*.

### Comparative genomic and MLST analysis

Comparative genomics used tools available in MAUVE version 2.3.1 [27]. The MAUVE Move Contigs tool was used to tile scaffolds generated by the *de novo* A5-miseq assembler against the reference *E. coli* APEC O78 finished genome. From this, the best alignment was chosen based on the highest weight score, an indicator of whether the predicted rearrangement exists, and lowest number of Locally Collinear Blocks (LCBs). Scaffolds that tiled against the finished APEC O78 genome were sorted and identified in this study as the subset representing the ‘core’ genome. The subset of scaffolds that did not align against the finished APEC O78 genome were designated the ‘accessory’ genome. The progressiveMauve module was used for comparative analysis of the genomes and to generate the figure. Regions of interest identified from whole genome comparisons were further analysed using iterative BLASTn and BLASTp searches.

The PubMLST (http://pubmlst.org/) database was used to identify the sequence type of the isolates using the Achtman *E. coli* MLST scheme [28] (http://mlst.warwick.ac.uk/mlst/).

## Results

### Whole genome sequence statistics and phylogenetics

De-novo assembly of the O157 SvETEC genome generated 236 scaffolds with 60-fold coverage and a predicted genome size of 5547789 nt. The N50 value for the assembly was 96352 nt. For O157:K88 734/3, de-novo assembly generated 226 scaffolds with 78-fold coverage, a predicted genome size of 5449663 nt and an N50 value of 91578 nt. In both cases, 50% of the respective genomes were assembled into the largest 18 scaffolds. Scaffolds were sorted into cohorts putatively representing the core and accessory genomes of O157 SvETEC and O157:K88 734/3 using the criteria described above. The O157 SvETEC core genome consisted of 96 scaffolds totalling 4789945 nt while the O157:K88 734/3 core genome comprised 94 scaffolds totalling 4809185 nt. The accessory genome of O157 SvETEC was 738983 nt in length and was represented in 112 scaffolds while the O157:K88 734/3 accessory sequence totalled 616231 nt in 97 scaffolds.

Phylogenetic analysis using the assembled genome sequences was performed to gain insight into the evolution of ETEC O157 isolates in Australian swine populations. *In-silico* identification of the genes used in the updated Clermont *et al.* phylotyping method [29] determined that O157 SvETEC and O157:K88 734/3 belonged to phylogroup C. This concurred with previously reported data [4].

**Figure 1:**
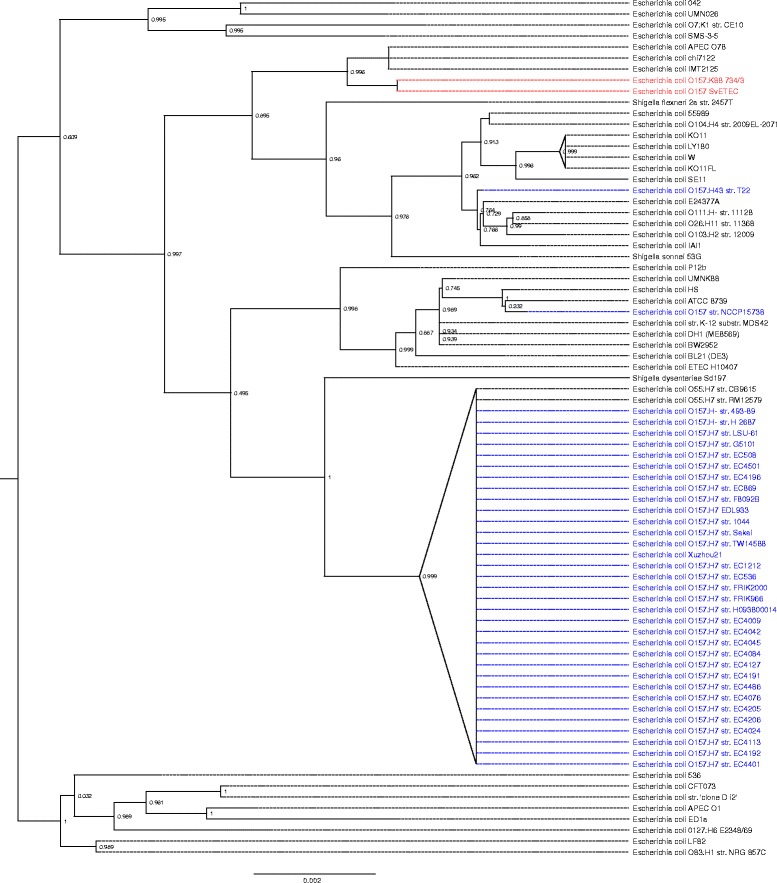
**A phylogenetic tree produced using PhyloSift.** The numbers located on each node is a confidence value between 0 and 1, with values near 1 indicating that the branch at that node exists with high probability. The scale is number of substitutions per site. *E. coli* O157 SvETEC and *E. coli* O157:K88 734/3 were placed into a single clade (red). The most closely related isolate was an avian pathogenic *E. coli* O78. Various completed and draft *E. coli* O157 genomes taken from the GenBank database clustered together (blue), with a confidence value of 1.

The PhyloSift phylogenetic analysis was performed to examine the swine isolates in the context of *E. coli* population structure. Figure 1 shows that neither *E. coli* O157 SvETEC nor *E. coli* O157:K88 734/3 clustered with the other *E. coli* serogroup O157 isolates and grouped together into a single clade with the most closely related completely closed reference genome being an avian pathogenic *E. coli* (APEC) of serogroup O78 [GenBank: CP004009]. Notably, the subclade included a variety of other isolates including *S. sonnei* 53G [GenBank:HE616528], known pathogenic *E. coli* serogroups such as O111 [GenBank:AP010960], O104 [GenBank:CP003301] and O26 [GenBank:AP010953] and a draft *E. coli* O157:H43 genome sequence [GenBank:AHZD02000001]. *E. coli* O157 draft and complete genomes as well as *E. coli* O55 genomes grouped together as a large clade in the tree, clustering with a confidence value of 1. The findings of our phylogenetic analysis were supported by eMLST analysis in which the O157 SvETEC and O157:K88 734/3 isolates represented a novel sequence type, ST4245 [4]. For comparative purposes, we also sequence typed APEC O78, the closest neighbour of the porcine O157 isolates and found it to be ST23, a member of the same clonal complex.

### Comparative genomics

**Figure 2:**
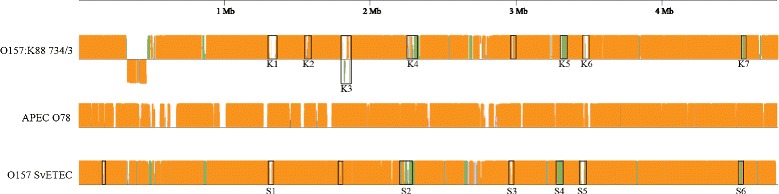
**Comparative progressiveMauve analysis between the core scaffolds of each whole genome sequence and a reference genome, the closely related** ***E. coli*** **APEC O78.** Predicted sequence homology among all three genome sequences is represented by orange regions. Green regions indicate sequence shared between the O157 core scaffolds only. Regions within black boxes are identified phage sequences and phage-associated regions. Sequence below the line of O157:K88 734/3 represents a predicted inversion, based on scaffold tiling to the APEC O78 reference.

The scaffolds representing the core O157 SvETEC genome had 4,741 predicted ORFs while the scaffolds representing the core O157:K88 734/3 genome consisted of 4,732 predicted ORFs. Both genomes displayed 94 RNA-encoding ORFs. A BLASTn comparison, facilitated by Mauve, of the O157 SvETEC and O157:K88 734/3 genomes was performed on the scaffolds representing the core genomes of both ETEC O157 against the finished reference genome of *E. coli* APEC O78 (Figure 1). Common features between the two ETEC O157 core genomes included an enterotoxin-encoding scaffold and the transposon Tn*7*, which were not present within the reference genome. Scaffolds carrying genes encoding enterotoxins LT and STb aligned to core genome sequences of both strains, likely due to an IS*911* gene aligning each scaffold to the same IS element in the APEC O78 genome sequence. BLASTn analysis of the LT/STb enterotoxin encoding scaffolds indicated similarity with pUMNK88_Ent [GenBank:CP002732] with both scaffolds exhibiting 99% sequence identity over 77% of the query sequence for O157 SvETEC and 79% for O157:K88 734/3. These data suggest that the enterotoxin genes reside on a plasmid. Variability between the core O157 genomes was mostly limited to phage associated regions scattered throughout each genome alignment. Six phage-associated regions, designated Phage Regions S1-S6, were identified within the O157 SvETEC core genome and seven, designated Phage Regions K1-K7 were identified in the O157:K88 734/3 core genome (Table 1 and Figure 2). Two of the six phage regions in the O157 SvETEC genome were identified to be intact prophages by PHAST whereas five of the seven O157:K88 734/3 phage regions were identified as intact prophages. The majority of these phage associated scaffolds were putatively composed of >85% phage and hypothetical gene content. Phage Region 1 of O157:K88 734/3 was most similar to a lambda phage and encoded the increased serum survival gene *bor*. Two identical phage-associated regions were shared by both O157 genomes. Phage Region 6 of O157 SvETEC and Phage Region 7 of O157:K88 734/3 were identical over 19.3 kb. Phage Region 4 of O157 SvETEC was 99% identical to Phage Region 5 of O157:K88 734/3. Two smaller regions in O157 SvETEC and one in O157:K88 734/3 were manually identified as phage associated regions and are represented by unlabelled boxes in Figure 2. In addition, the scaffold encoding the K88 adhesin operon in the O157:K88 734/3 genome also encoded several phage-associated genes that were lacking in the homologous O157 SvETEC scaffold. As such, this scaffold was aligned into the O157:K88 734/3 core genome due to these phage-associated genes. Variation between all three genome sequences was also observed around the APEC O78 O-antigen biosynthesis genes.

**Table 1:** **Phage associated regions identified by PHAST**

**Strain**	**Region**	**Concatenated** ^**1**^ **Boundaries (bp)**	**Size (kb)**	**Ordered scaffold numbers**	**PHAST Annotation**	**Annotation Identity** ^**2**^ **(Phage Identity** ^**3**^ **)**	**PHAST Prediction** ^**4**^
*E. coli* O157 SvETEC	S1	1316571-1334538	17.9	83, 114, 2	Stx2 converting I NC_003525	24% (92%)	Incomplete
	S2	2204749-2280987	76.2	59, 58, 107, 53, 39	mEp460 NC_019716	22% (90%)	Intact
	S3	2966791-2982739	15.9	32, 81, 1	P1 NC_005856	50% (85.7%)	Incomplete
	S4	3287635-3331585	43.9	1, 11	TL 2011b NC_019445	78.57% (98.2%)	Intact
	S5	3449037-3477233	28.1	44, 70, 14	P1 NC_005856	39.39% (84.8%)	Incomplete
	S6	4542510-4561835	19.3	31	APSE 2 NC_011551	15.78% (47.3%)	Incomplete
*E. coli* O157:K88 734/3	K1	1322304-1357135	34.8	67, 55, 141, 6	lambda NC_001416	54.76% (97.6%)	Intact
	K2	1555282-1584506	29.2	34, 83, 58	Fels 2 NC_010463	57.14% (100%)	Incomplete
	K3	1810316-1849764	39.4	19, 41	mEp460 NC_019716	17.94% (92.3%)	Intact
	K4	2254746-2314331	59.5	54, 65, 94, 98, 132, 136, 38	mEp460 NC_019716	29.11% (92.4%)	Intact
	K5	3315282-3359241	43.9	2, 23	TL 2011b NC_019445	78.18% (98.1%)	Intact
	K6	3476711-3497698	20.9	30	Fels 2 NC_010463	86.2% (100%)	Intact
	K7	4558357-4577682	19.3	7	APSE 2 NC_011551	15% (45%)	Incomplete

^1^Identified regions were generally over multiple scaffolds but were identified as concatenated sequence. ^2^Percent identity of the region compared to the PHAST annotation. ^3^Percentage of identified ORFs that encode phage or hypothetical genes. ^4^PHASTs prediction of whether each prophage encodes the genes necessary for lysogeny.

The accessory genome scaffolds of O157 SvETEC and O157:K88 734/3 were examined for ORFs. O157 SvETEC comprises 1008 predicted ORFs over 112 scaffolds including 8 RNA predictions, whereas O157:K88 734/3’s comprises 851 predicted ORFs over 97 scaffolds including 8 RNA predictions. To further assess variability between the two genomes a comparative BLASTp analysis of the predicted amino acid sequence of the accessory genome ORFs of O157 SvETEC and O157:K88 734/3 was performed (Additional file 1: Table S1). Several scaffolds and consequently their predicted peptide sequences were determined to be unique to O157 SvETEC. The majority of genes common between the accessory genomes were plasmid related and included *tra* operon genes associated with IncF and IncI plasmids, and hypothetical proteins. Fragments of complex antimicrobial resistance loci (CRL) were also shared between the two accessory genomes including a Tn*21*-like mercury resistance operon and scaffolds associated with clinical class 1 and class 2 integrons. Several phage-containing or phage gene-associated scaffolds were also identified within the accessory genomes. Two approximately identical scaffolds of 29133 nt and 29044 nt respectively for O157 SvETEC and O157:K88 734/3 were identified as most similar to Enterobacteria phage P1 (NC_005856) but were predicted to be incomplete by PHAST. Both accessory genomes encoded plasmid transfer genes relating to the plasmid incompatibility groups F and I, however the O157 SvETEC accessory scaffolds also encoded IncH plasmid-associated genes. BLASTn analysis of the 66 kb (Scaffold 26) encoding IncH1 plasmid-associated genes identified seven GenBank entries each with 96-99% query coverage and 98-99% identity with the scaffold. Each entry was of a plasmid and included *Serratia marcescens* plasmid R478 [GenBank:BX664015], *Salmonella enterica* subsp. *enterica* Serovar Cubana str. CFSAN002050 plasmid [GenBank:CP006056] and *Escherichia coli* APEC O1 plasmid pAPEC-O1-R [GenBank:DQ517526]. Several other scaffolds within the O157 SvETEC accessory genome also aligned with high identity to these plasmids. These scaffold generally encoded hypothetical proteins with the exception of a copper/heavy metal (*cus/czc*) resistance operon (Scaffold 43) and a tellurium/tellurite (*ter*) resistance operon (Scaffold 35).

The O157 SvETEC accessory genome was also characterised by the presence of a Tn*7*-like transposon operon, hygromycin resistance, the enterotoxin STa and a second allele of STb, all of which were lacking in the O157:K88 734/3 accessory genome. A 17.8 kb region of another accessory O157:K88 734/3 scaffold was identified to contain an incomplete phage most similar to Enterobacteria phage P1 (NC_005856). Further BLASTn analysis suggested both ETEC O157 genomes contained only a single copy of the P1-like phage, which has been split amongst various core and accessory scaffolds due to the reference tiling. Aside from these specifics, much of the variation between the accessory genomes of the two strains lies in plasmid associated hypothetical proteins, mobile elements and CRL-associated scaffolds.

The O-antigen biosynthesis genes within both ETEC O157 draft genome sequences were split across several scaffolds making detailed analysis difficult. Despite this, high synteny was observed between the O157-antigen encoding scaffolds with both isolates encoding the Sakai-type *rfbE* gene (gene truncated at 1032 bp by a scaffold break in O157:K88 734/3) and also containing the genes *yoeB* and *yefM* identical to the *E. coli* strain PV00-24 O-antigen biosynthesis gene cluster [GenBank: AB602253.1][8].

### Virulence factors

As both the ETEC O157 pathogens belong to the novel ST4245 sequence type we were particularly interested to identify potential virulence determinants within their genomes. Those we identified through sequence analysis are detailed in Table 2, including a variety of adhesin and toxin genes and their associated operons. Both O157 SvETEC and O157:K88 734/3 were positive for the enterotoxins LTp (*eltAB)* and STb (*estB*) whereas the heat stable enterotoxin STa (*estA*) was only identified in O157 SvETEC. A further distinction highlighted by sequence analysis was that a second allele of *estB* was present in a separate scaffold within the O157 SvETEC genome. This allele differed by a single nucleotide polymorphism and was identical to the STb gene of *Escherichia coli* F18+ strain EC2173 plasmid pTC1 [GenBank:CP000913]. The *fliC* gene was, in both isolates, the H19 allele.

**Table 2:** **Virulence genes associated with pathogenicity present in ETEC O157**

**Type**	**Gene**	**O157 SvETEC**	**O157:K88 734/3**	**Details**
**Adhesins**	*faeG*	+	+	K88 (F4) Fimbrial adhesin
	*fimH*	+	+	Type 1 fimbriae, D-Mannose-specific adhesin
	*csgG*	+	+	Facilitator of fibronectin-binding curli assembly
	*eaeH*	+	+	Highly conserved adhesin
	*sfmA*	+	+	Fimbrial-like adhesin
	*ecpA*	+	+	*E. coli c*ommon pilus
	*mat*	+	+	*mat* operon
**Toxins**	*east*1	+	+	Enteroaggregative *E. coli* heat-stable enterotoxin
	*hlyA*	+	+	α-Hemolysin
	*hlyE*	+	+	Hemolysin E
	*eltB*	+	+	Heat-labile enterotoxin B subunit
	*eltA*	+	+	Heat-labile enterotoxin A subunit
	*estA*	+		Heat-stable enterotoxin a
	*estB*	+	+	Heat-stable Enterotoxin b
	*estB*	+		Heat-stable Enterotoxin b (second allele)
**Siderophores**	*fyuA*	+	+	Iron uptake receptor
	*irp*2	+	+	Iron repressible protein
**Other**	*bor*		+	Serum survival
	*traT*	+	+	Surface exclusion and serum survival
	*fliC*	+	+	Flagellin structural protein, H antigen determinant (H19 allele)

### Antimicrobial resistances

Multiple antibiotic resistances were observed in the phenotypic analysis of O157 SvETEC and O157:K88 734/3. Both isolates were resistant to ampicillin, nalidixic acid, sulphafurazole, trimethoprim, tetracycline and neomycin. Each isolate displayed distinct additional resistance phenotypes against apramycin and chloramphenicol for O157 SvETEC and against cephalexin and cefoxitin for the O157:K88 734/3 isolate [4]. Various genes known to provide these antimicrobial resistances were identified within the genome sequences (Table 3). The *gyrA* sequence of both isolates was determined to contain a single S83L amino acid change compared with the *E. coli* K12 substrain MG1655 *gyrA* gene.

**Table 3:** **Antibiotic and heavy metal resistance genes identified within ETEC O157**

**Resistance gene/Operon**	**O157 SvETEC**	**O157:K88 734/3**	**Phenotypic function/Resistance**	**Resistance observed**
*intI*1	+	+	Class 1 Integron Integrase	
*intI*2	+	+	Class 2 Integron Integrase	
*bla*_TEM_	+	+	Ampicillin	+
*ampC*	+	+	Cephalosporins	O157:K88 734/3 only
*gyrA*	+	+	Quinolones	+
*aphA*1	+	+	Aminoglycosides	+
APH(4)-IA	+		Hygromycin B	O157 SvETEC only
*strAB*	+	+	Streptomycin	+
*aadA*	+	+	Streptomycin and Spectinomycin	+
*dfrA*	+	+	Trimethoprim	+
*aacC*	+		Apramycin	O157 SvETEC only
*sul1*	+	+	Sulphonamides	+
*sul2*	+	+	Sulphonamides	+
*sul3*	+		Sulphonamides	+
*macAB*	+	+	Macrolides	
*tet* operon	+	+	Tetracycline	+
*cmlA*	+		Chloramphenicol	O157 SvETEC only
*emr* operon	+	+	RND Efflux Pump	
*gadX*	+	+	Acid Resistance Regulator	
*mdt* operon	+	+	RND Efflux Pump	
*cus* operon	+	+	Heavy Metals	
*mer* operon	+	+	Mercury	
*ter* operon	+		Tellurium	

## Discussion

In this study, we were able to characterise the evolutionary background of two non-EHEC *E. coli* O157 strains, and identify genetic differences between these two ETEC strains known to adopt different pathogenic strategies; *E. coli* O157:K88 734/3 associated with neonatal diarrhoea and *E. coli* O157 SvETEC associated with severe pre- and post-weaning disease. Our analysis demonstrated an evolutionary contrast between ETEC O157 and EHEC O157 and identified various small differences in toxin and antimicrobial resistance gene content between the two ETEC O157 isolates.

### Molecular evolution of ETEC O157 strains

The PhyloSift analysis demonstrated that the O157 SvETEC and O157:K88 734/3 ETEC isolates are most closely related to an APEC O78 isolate. This is interesting because previous work on APEC O78 isolates has reported such isolates to be closely related to human ETEC isolates [30]. PhlyoSift analysis also demonstrated a degree of relatedness between the ETEC O157 isolates, *E. coli* W and enterohaemorrhagic *E. coli* O111:H^−^. These results highlight the similarity of various *E. coli* pathotypes and serogroups when observed from a genomic perspective. In doing so these results further stress that clonal ancestry can play a minor role in predicting pathogenesis compared with the lateral acquisition of virulence factors. The PhyloSift analysis, which utilised the DNA sequences of 37 conserved marker genes, also demonstrated a contrast between the genetic relatedness of most *E. coli* O157:H7 genomes from the GenBank database and our ETEC O157:H19 genomes. Two O157 genomes proved exceptions to this observation; *E. coli* O157:H43 str. T22 from Hungary [31] and O157:H42 str. NCCP15738 from Korea (GenBank:ASHB01000001.1). Clustering of *E. coli* O157:H7 isolates and the divergence of non-H7 strains in the phylogenetic analysis supports the idea that H-antigen typing may be a useful indicator of *E. coli* O157 lineage. In addition, the atypical (*stx*- and *eae-*negative) O157:H43 str. T22 encode CDT and long polar fimbriae [29], which underscores how O157 isolates carrying different H-antigens may carry different repertoires of virulence genes.

Observations of *E. coli* relatedness similar to these have been made previously in relation to the parallel evolution of EHEC pathogenesis within the distantly related serogroups such as O157, O26 and O111 [32], where pathogenesis was mediated by similar laterally acquired elements such as plasmids and prophages. This phylogenetic divergence also suggests genetic recombination played a role in ETEC O157 development, with the ETEC O157 isolates encoding the “Sakai-type” O157 O-antigen biosynthesis gene cluster [8]. The acquisition of this biosynthesis gene cluster is an event known to have also shaped the evolution of enterohaemorrhagic *E. coli* O157 [33].

Multi-Locus Sequence Typing analysis of the O157 SvETEC and O157:K88 734/3 genomes supported their close evolutionary relationship to each other and to the ST23 complex which includes APEC O78. In the MLST database *E. coli* ST23 isolates have been sourced from avian, porcine, human and bovine hosts. Enterotoxigenic *E. coli* O157 strains of porcine origin from different geographic locations have been submitted to the MLST database as ST90 (ST23-complex) although we could find little mention or analysis of these strains in the literature.

### Comparison of ETEC O157 core and accessory genomes

Analysis of scaffolds representing the core genome from O157 SvETEC and O157:K88 734/3 highlighted the close relationship of these strains. Genetic differences were largely associated with the acquisition of prophage-associated sequences. Some prophages were common between the two isolates suggesting they may have been acquired prior to an evolutionary divergence. One phage in O157:K88 734/3 contained the *bor* gene which is known to be associated with increased serum survival. The *bor* gene has a lambda phage origin and is generally associated with avian extraintestinal pathogenic *E. coli* [34].

The variability of the accessory genomes for each isolate is mediated mostly by plasmid sequences, particularly IncH1 plasmid genes and heavy metal resistance operons, indicating that the differences in pathogenesis between these two isolates is essentially associated with plasmid content. Attempts to purify plasmids from our isolates using various plasmid extraction kits and standard alkaline lysis methods were unsuccessful. It is possible that the plasmids are extremely large, beyond the capability of the extraction methods used, or they are embedded in the chromosomes of these isolates. We were unable to confirm the presence of genomic islands in our analysis because of the reference genome based tiling exercise employed to separate the subset of scaffolds potentially encoding the core genome and accessory genomes. Large mass plasmids encoding resistance to multiple antibiotics in *E. coli* isolated from the faeces of food animals in Australia have been described [35,36] and as such may play a role in the pathogenesis of these isolates. Heavy metals are used frequently in pig production as antimicrobials and the identification of copper and mercury resistance loci in both strains and tellurium resistance in O157 SvETEC are likely provide significant survival advantages to these isolates. Antimicrobial resistance genes, discussed below, provide further advantages to these isolates. Such genes are often found on plasmids carrying mercury resistance transposons that serve as conduits for the lateral transfer of such genes [37,38].

### Virulence factors

*E. coli* virulence factors that are routinely screened for globally [4,39,40] were identified in the O157 SvETEC and O157:K88 734/3 genomes. These included the porcine ETEC specific K88 fimbrial adhesin, and several adhesins commonly seen in other pathotypes of *E. coli*. The *fimH* gene, which encodes a mannose-specific adhesin in extraintestinal pathogenic *E. coli* [41] has also been observed in commensal *E. coli* isolates or porcine origin [42]. BLAST analysis identified various adhesins, however most of these are common to *E. coli* isolates and have no definitive role in pathogenicity. The primary difference between the virulence profiles of the O157 SvETEC strain and the O157:K88 734/3 strain is that the latter lacks the heat-stable enterotoxin a (STa) and a second copy of STb. Links between the enterotoxins present in an isolate and the form of pathogenesis caused by ETEC have been described previously [43,44] and these differences may impact pathogenesis. Previous work has also shown that the pTC plasmid-like variant of STb, which is homologous to the extra STb allele encoded in O157 SvETEC, is not enterotoxigenic *in vivo* [45,46].

Other virulence associated genes identified in the O157 SvETEC and the O157:K88 734/3 genomes included the flagellin structural protein *fliC*. The presence of the *fliC* gene, which encodes the H19 flagellar antigen [47], is important. Previous studies have demonstrated a correlation between the expression of flagellum and the K88ac fimbrial antigen [48] where the expression of both *fliC* and *faeG,* the major subunit of the K88 adhesin, correlates with adhesion to porcine epithelial cells *in vitro*. The characterisation of the flagellum also allows us to identify the serotype of both isolates as *E. coli* O157:H19:K88. Pathogenic *E. coli* of the serotype O157:H19 have been very sparingly reported [9] and studies involving isolates of this serotype have focussed on triclosan tolerance [49,50]. These studies do indicate that O157 strains have a capacity to adapt to multiple pathogenic lifestyles, having been identified as verocytotoxigenic, enterotoxigenic and enteropathogenic *E. coli*.

Another factor that appears to influence ETEC pathogenicity is the host’s intestinal development and environment. Environmental conditions have an impact on enteroheamorrhagic *E. coli* pathogenesis and the regulation of virulence factor expression [51]. A similar situation has been observed *in vitro* for ETEC, with LT expression modified by pH [52]. Also, it has been demonstrated that the porcine intestinal epithelium can develop a resistance to F18-mediated adherence [53] and the stress of early weaning reduces the ability of piglets to cope with ETEC infection [54]. This data indicates that whilst it is important to characterise pathogenic *E. coli*, host characteristics and the intestinal environment may play a role in altering pathogenicity and account for the variation in disease severity between these isolates given their highly similar virulence factor content.

### Antibiotic resistance

The greatest disparity between the O157 SvETEC and O157:K88 734/3 isolates is their antibiotic resistance. Both have quite comprehensive resistance profiles being resistant to beta-lactams, quinolones, aminoglycosides and cephalosporins. In context, one of the most important antimicrobial resistance genes in either strain is *aphA1* which confers resistance to neomycin and kanamycin, with neomycin being a drug of choice for the treatment of infection during pig development within Australian pig farms. The majority of the observed resistance phenotypes have been accounted for including a known nalidixic acid resistance mutation in the DNA gyrase gene *gyrA*. Large complex antimicrobial gene resistance loci centred around both class 1 and class 2 integrons were identified within these genome sequences, however the prevalence of the insertion sequence IS*26* in these regions precluded the automated assembly of the loci.

## Conclusion

O157 SvETEC and O157:K88 734/3 appear to be part of a poorly characterised lineage of *E. coli* O157, which differs significantly from EHEC O157. Differences in pathogenicity between the strains may stem from differences in acquired virulence factors, antimicrobial resistance, and phage related genes (the majority of which are uncharacterised hypotheticals) with *E. coli* O157 SvETEC encoding a larger toxin repertoire compared to O157:K88 734/3. Further studies with these strains will focus on the assembly of antibiotic resistance gene loci and their association with mobile genetic elements.

## Additional file

Additional file 1:
**A spreadsheet containing a BLASTp comparison of all identified open reading frames in the accessory genomes of both O157 SvETEC and O157:K88 734/3 draft genome sequences.** Data includes scaffold numbers, ORF annotations and BLASTp homology as a percentage.
